# Socioeconomic differences in informal caregiving in Europe

**DOI:** 10.1007/s10433-021-00666-y

**Published:** 2021-12-14

**Authors:** Nekehia T. Quashie, Melanie Wagner, Ellen Verbakel, Christian Deindl

**Affiliations:** 1 Department of Social Sciences, TU Dortmund University, Dortmund, Germany; 2 Max Planck Institute for Social Law and Social Policy, Munich Center for the Economics of Aging (MEA), Munich, Germany; 3 Department of Sociology, Radboud University, Nijmegen, The Netherlands

**Keywords:** Informal caregiving, Socioeconomic inequality, Europe, Cross-national

## Abstract

Disclosing socioeconomic differences in informal care provision is increasingly important in aging societies as it helps to identify the segments of the population that may need targeted support and the types of national investments to support family caregivers. This study examines the association between individual-level socioeconomic status and informal care provision within the household. We also examine the role of contextual factors, income inequality, and the generosity of social spending, to identify how macro-level socioeconomic resource structures shape individuals’ provision of care to household members. We use pooled data from the Survey of Health, Ageing and Retirement in Europe (SHARE, waves 1, 2, 4, 5, 6) and the English Longitudinal Study of Ageing (ELSA, waves 2, 3, 4, 6, 7). Poisson regression multilevel models estimate the associations between household socioeconomic status (education, income, and wealth), and country socioeconomic resources (income inequality and social spending as a percentage of GDP), and the likelihood of older adults’ informal care provision within the household. Results indicate that lower individual socioeconomic resources—education, income, and wealth—were associated with a higher incidence of older adults’ informal care provision within the household. At the macro-level, income inequality was positively associated while social spending was negatively associated with older adults’ care provision within the household. Our findings suggest that socioeconomically disadvantaged groups are more likely to provide informal care, which may reinforce socioeconomic inequalities. At the national level, more equitable resource distribution and social spending may reduce intensive family caregiving.

## Introduction

In the light of the ongoing demographic aging in most Western countries, inequality in care is an important topic. Formal care is often costly and not always fully covered by health insurance or social security programs. This can lead to an unequal distribution of the use and provision of formal and informal care. To date, socioeconomic inequality in informal care *provision* has been inadequately addressed in social research, despite the growing literature on socioeconomic status (SES) differences in informal care *use* and cross-national variation in the relationship between socioeconomic inequality and informal care use (e.g., Albertini and Pavolini 2017; Broese van Groenou et al. 2006; Rodrigues et al. 2018). The existing research on socioeconomic inequality in informal care provision has largely focused on caregivers’ employment situation or the consequences of caregiving for employment, especially among women (Vlachantoni 2010; Moussa 2019). We will add to research by analyzing the associations between informal care provision and three different SES measures: education, income, and wealth in a European welfare state context. The inequality focus helps to identify which individuals are most likely to take up informal care and thus to face its consequences. We focus on informal adult care within the household, which is typically provided to the caregiver’s partner or parents and is normally an intensive form of support with adverse health consequences for informal caregivers (Kaschowitz and Brandt 2017). By focusing on this form of care, we make sure to capture intensive care provision where inequalities will show most prominent.

In addition to these micro-level inequalities, we examine the influence of social inequality and social spending at the country level. Social inequality has an undeniable impact on family relations (Deindl and Brandt 2015, 2019). Arguably, social inequality at the macro-level reduces solidarity within societies (Wilkinson and Pickett, 2009) and it is likely that familial solidarity also suffers. For instance, generous government social spending likely shapes the necessity and opportunity of people to provide care for their dependent loved ones (Brandt et al. 2009; Verbakel 2018). Understanding how country-level indicators shape informal care provision within the household is important for identifying which types of investments may be useful in reducing the strain put on informal caregivers.

We will therefore analyze the role of the macro-context on caregiving by introducing income inequality and social spending at the country level as further explanatory variables. We use the *Survey of Health, Ageing and Retirement in Europe* (SHARE), the *English Longitudinal Study of Ageing* (ELSA) as micro-level data, and Eurostat and World Bank data on country characteristics, to examine socioeconomic differences in the provision of informal care in Europe with a multilevel approach. The incorporation of these datasets allows for cross-country comparisons by their degrees of income inequality and social spending (Esping-Andersen 1999; Saraceno 2016). This will enable us to answer the following research questions: Are socioeconomic differences (in education, income, and wealth) related to the provision of informal care? Can differences in the provision of informal care across Europe be explained by differences in income inequality and social spending?

## Socioeconomic differences in informal care provision

The care needs of dependent adults may be met by formal care arrangements (usually in a nursing home or with professional nurses in their own home), informal care, or a combination of formal and informal care. Whether an individual provides informal care to their dependent household member depends on the needs, opportunities and means, and preferences and beliefs of both the care user (Andersen and Newman 2005) and the informal caregiver (Cooney and Dykstra 2011; Brandt and Deindl 2013; Broese van Groenou and de Boer 2016; Haberkern and Szydlik 2010; Walker et al. 1995). These prerequisites to care also differ between socioeconomic groups in several ways, as we will discuss below.

With respect to needs for care, research shows that lower socioeconomic groups have worse health, potentially due to unfavorable working and living conditions and harmful health behaviors (e.g., Arber and Ginn 1992). As a result, the need for care is higher in families with a lower socioeconomic status, and thus, the likelihood to provide informal care to a dependent household member is higher for individuals with lower socioeconomic resources.

Opportunities and means are generally more favorable among higher SES individuals, and research suggests that they generally use these resources to avoid informal care. Research on SES differences in the use of care shows that care dependents with higher income and wealth are more likely to access and pay for community care (Floridi et al. 2020; Rodrigues et al. 2018), which in return means that their family members will provide less informal care. Research comparing SES indicators, specifically income and wealth, has shown that informal care use was negatively associated with both measures with income showing stronger associations than wealth (Rodrigues et al. 2018). From the point of view of the caregiver, socioeconomic status is negatively associated with informal care provision, especially for women (Henz 2006). This could be a reinforcing process. Those with little employment obligations can more easily pick up informal caregiving as they generally feel fewer time conflicts, but research also shows that caregiving women are more likely to reduce their working hours or stop working completely, which may negatively affect their socioeconomic position (Moussa 2019).

Family caregiving norms are found to be stronger among lower SES groups (Daatland and Herlofson 2003). Provided that family members agree with norms that they should help each other when in need, the potential caregiver may not hesitate to provide care when a family member is in need, and the person in need of care may feel less reluctant to rely on informal carers, thereby encouraging informal care provision (Broese van Groenou and de Boer 2016). Also with respect to preferences and beliefs, we expect higher informal care provision rates among lower SES individuals.

Overall, given the SES differences in needs, opportunities and preferences, we expect to find that compared to non-caregivers, caregivers have lower SES in terms of education, income, and wealth.

### Country differences in informal care provision

Besides individuals’ socioeconomic positions, whether one provides informal care depends on the macro-level context such as a country’s income distribution and social policies (Broese van Groenou and de Boer 2016). Previous studies suggest that inequality at the national and regional levels (Deindl and Brandt 2015, 2019) leads to less societal involvement including care provision. The argument is that social solidarity is weakened by social inequality (Wilkinson and Pickett 2009) and it is likely that familial solidarity also suffers, resulting in lower willingness to provide care to dependent family members.

Welfare states have different policies in favor of formal or informal provision of care (see Saraceno 2016 for a detailed discussion). There are different arguments regarding the direction in which generous welfare states shape individuals’ informal care provision (Verbakel 2018). Formal and informal care may be seen as substitutes, whereby high levels of social spending may lower the need for informal care. Alternatively, high levels of social spending facilitate helping behavior by reducing financial or time barriers. Moreover, a supportive state underscores the norm to help others in need. Empirical support is found for an in-between view, which argues that there is a specialization between the state and the family (e.g., Brandt et al. 2009; Verbakel 2018). Specialization here means that in countries where the welfare state is more generous and provides higher levels of formal support, the family is less involved in informal *care* but focuses their support on *non-care*-related activities (Brandt et al. 2009). Likewise, generous state support goes together with less *intensive* informal caregiving, but with a higher overall rate of informal caregivers (Verbakel 2018). The underlying assumption is that the state assumes responsibility for more regular, and intensive, support while the family is more involved in sporadic support (see Brandt 2013 for further details).

In sum, we expect higher levels of societal inequality to be associated with lower family care provision due to lower solidarity among individuals and higher social spending to be associated with lower family care due to higher state responsibility for intensive care.

## Method

### Data and sample selection

We analyzed pooled data from the *Survey of Health, Ageing and Retirement in Europe* (SHARE), from 2004 to 2015 (release 7.0.0), and the *English Longitudinal Study of Ageing* (ELSA) from 2002 to 2015. Both datasets have representative samples of individuals aged 50 and older across England, Continental Europe, and Israel (Börsch-Supan et al. 2013; Steptoe et al. 2013), and are designed for cross-national comparability. Both studies biannually resurvey the respondents and their cohabiting partners by computer-assisted personal interviews. Respondents answer questions on their family, economic, and health situation and the care given to family and friends inside and outside the household.

Our pooled dataset used the first interview of respondents in SHARE or ELSA. Table 1 provides an overview of the specific waves used in our analyses, which also provide comparable survey years from 2004 to 2015. Data from ELSA wave one (2002–2003) were excluded because the location of care recipients (whether inside or outside of the household) was only available from wave two onwards. Therefore, we used ELSA wave 2 as our baseline. Data from waves five (2010–2011) and eight (2016–2017) were also excluded as new respondents represent partner interviews rather than core ELSA participants. Regarding SHARE, we excluded data from the third and seventh waves due to their focus on respondents’ life histories (SHARELIFE). Our pooled data also excluded Israel due to our focus on European countries.

**Table 1:** Overview of the SHARE AND ELSA waves, and the respective years, included in our analyses

Waves	ELSA	SHARE
1	n.i.	2004–2005
2	2004–2005	2006–2007
3	2006–2007	n.i.
4	2008–2009	2011–2012
5	n.i.	2013
6	2012–2013	2015
7	2014–2015	n.i.

*n.i.* not included

Our pooled sample included data from 125,958 respondents from 21 European countries across Northern (Denmark and Sweden), Western (Austria, Belgium, England, France, Germany, Ireland, the Netherlands, Luxembourg, and Switzerland), Southern (Portugal, Spain, Italy, and Greece), and Eastern (Estonia, Croatia, Czech Republic, Hungary, Poland, and Slovenia) Europe. The analyses focused on care provision within the household, as within-household care is the most intensive form of care. Moreover, questions regarding care provision inside the household were largely consistent across all the SHARE waves and comparable to ELSA, in contrast to questions on care provision outside the household. We excluded respondents living alone (*n* = 24,489), and respondents only living with children younger than 18 years (*n* = 574). Due to missing data on the variables included in our analyses (*n* = 3,023), our analytic sample for caregiving within the household included 97, 872 respondents with complete data on all variables. Country-level data drawn for each year of the interview, from Eurostat and the World Bank, supplemented the individual-level data.

## Dependent variable

The dependent variable was informal care provision inside the household. In both studies, respondents reported whether they provided care to someone and their relationship to the recipient (e.g., partner, parent, parent-in-law, child, grandchild, other relatives, friend or neighbor). In ELSA waves 2 to 4, questions on care provision proceeded in two steps. First, respondents were asked, “*Did you do any of the following activities during the past month?”* Those who identified caring for someone as one of their activities in the past month were then asked, *“Did you look after someone in the past week (including your partner or other people in the household)? By ‘look after’ we mean the active provision of care.”* In waves 6 and 7, however, the question on care provision in the past week was unrelated to whether respondents indicated caring for someone as one of their activities in the past month. In waves two to four, respondents who did not care for someone within the past month were included as non-caregivers as they, by definition, also did not care for someone in the past week. In waves two to seven, respondents who indicated caring for someone in the past week were asked whether they lived with the care recipient (yes or no). Therefore, within-household caregivers in ELSA represented those who provided care in the past week and indicated they lived with the recipient.

In SHARE, we measured care provision inside the household based on the question, “*Is there someone living in this household whom you have helped regularly during the last twelve months with personal care, such as washing, getting out of bed, or dressing? By regularly we mean daily or almost daily during at least three months. We do not want to capture help during short-term sickness of family members.”* We categorized affirmative responses as care provision inside the household. In both surveys, non-caregivers within the household referred to respondents who did not provide care within the household and might include respondents who provided care outside the household. The questions regarding care provision differed somewhat between ELSA and SHARE. Separate analyses for SHARE and ELSA and the consistent prevalence of care between both surveys did not point to any problems to combine both surveys.

## Independent variables

### Individual-level measures

Our main individual-level independent variables included three indicators of household socioeconomic status: education, income, and wealth. The household’s highest level of *education* is a categorical measure that represents the highest education level of either the respondent or their partner. We derived our coding from the International Standard Classification for Education (ISCED) classification: low (ISCED 0–2 in SHARE, no educational qualifications in ELSA), medium, reference group (ISCED 3 and 4 in SHARE, higher education below a degree, GCE A and O Levels or CSE other grade in ELSA), and high (ISCED 5 and 6 in SHARE, higher education diploma or certificate in ELSA).

Material status was measured by income and wealth. We followed the typical approach to measure inequality in income and wealth by using a relative approach for income and an absolute approach for wealth. We used a harmonized measure of total yearly couple *income* provided by the Gateway to Global Aging (please see the Gateway to Global Aging guide Beaumaster et al. 2019; Gateway to Global Aging Data Team 2020; Lee 2015). In ELSA, we converted income to Euros using the average annual exchange rate for the respective survey years (UK Office of National Statistics 2019). We adjusted total income for household size using an equivalence scale that assigns a value of 1 to the household head and 0.5 to each additional member. We then categorized this equivalized income based on a household’s position relative to the median income in each wave and country. The categories included: (1) poor (below 50% of median income), (2) low middle income (50% of median income to median income), (3) middle medium income, reference group (median income to 200% of median income), (4) high middle income (200% to 300% of median income), and (5) high income (above 300% of median income). *Wealth* (also converted to Euros in ELSA), which includes the net sum of financial (e.g., savings, investments, minus liabilities) and real assets (e.g., value of housing minus mortgage, other physical wealth), was categorized as (1) debts, negative wealth, (2) 0–49 999 (reference group), (3) 50 000 to 99 999, and (4) 100 000 or more.

## Country-level measures

We considered two country-level characteristics: income inequality and welfare spending. We used the Gini coefficient of *income inequality* in the general population to measure country-level income inequality. This value ranges from 0 to 100 whereby 0 represents complete equality and 100 complete inequality, regarding the income distribution of households in a given country. The Gini coefficient values were drawn from the United Nations University—World Institute for Development Economics Research (UNU-WIDER) World Income Inequality Database (UNU-WIDER WWID 2019), which also draws from the World Bank. National level of *social spending* was measured by total social protection expenditure as a percentage of the country’s GDP, hereafter referred to as social spending, provided by Eurostat (Eurostat 2020). Social spending encompasses interventions from public or private bodies to support individuals and households burdened by risks or needs that arise related to sickness, disability, survivors, family/children, old age, unemployment, housing, or general social exclusion. We averaged the data points across all available years, for each country, to create one score per country. Thus, the indicators represent the mean Gini coefficient and social expenditure at the country level.

Covariates included age (continuous), gender (male = 0, female = 1), household size (continuous), employment status (0 = not working, 1 = full-time, 2 = part-time), limitations with instrumental activities of daily living (iadl) as an indicator of physical health (0 = No iadl limitations, 1 = 1 or more limitations), and interview year (2004–2005, 2006–2007, 2008–2012, and 2013–2015). We combined the interview years to reflect comparable waves of SHARE and ELSA.

## Analysis

Descriptive statistics of the individual-level characteristics of our sample as well as care provision within the household, individual socioeconomic indicators, and the country-level indicators for each country within our study are presented in Tables 2 and 3, respectively.

**Table 2:** Description of the care provision within the household by individuals’ caregiving status

	Non-caregivers within the household	Caregivers within the household	Total
	*n* = 90,667	*n* = 7,205	*n* = 97,872
Variables	%, mean (sd)	%, mean (sd)	%, mean (sd)
*Individual level*			
*Household education*			
Medium	40.74	38.15	40.55
Low	31.29	40.64	31.98
High	27.97	21.21	27.47
*Couple income*			
Middle medium income	38.20	34.70	37.94
Poor	15.13	17.31	15.29
Low medium income	29.68	35.70	30.12
High medium income	9.82	6.32	9.56
High income	7.18	5.98	7.09
*Household wealth*			
0 to 49 999	21.80	29.85	22.40
Debt	2.64	3.22	2.69
50 000 to 99 999	12.57	13.91	12.67
100 000 or more	62.98	53.02	62.25
Age	62.88(9.32)	65.41(10.33)	63.07(9.42)
Household size	2.50(0.92)	2.58(0.99)	2.51(0.92)
*Gender*			
Men	49.94	41.72	49.34
Women	50.06	58.28	50.66
*Employment*			
Not working	64.72	77.53	65.66
Full-time	25.87	16.03	25.14
Part-time	9.41	6.44	9.19
*IADL limitations*			
No iadl limitations	86.47	75.75	85.68
1 + iadl limitations	13.53	24.25	14.32
*Interview year*			
2004–2005	27.43	22.40	27.06
2006–2007	13.48	13.37	13.48
2008–2012	30.50	34.80	30.82
2013–2015	28.58	29.44	28.65
*Country level*			
Mean Gini coefficient			31.20(3.15)
Mean Social Expenditure, % GDP			23.91(4.26)

^a^ sd = standard deviation. ^b^ Interview years 2004–2005 represent SHARE wave 1 and ELSA wave 2, Years 2006–2007 represent SHARE wave 2 and ELSA wave 3, Years 2008 to 2012 represent SHARE wave 4, and ELSA waves 4, Years 2013–2015 represent SHARE waves 5 and 6, and ELSA waves 6 and 7. ^c^ Means of country-level indicators are derived from our sample of 21 countries

**Table 3:** Percentage distribution of individual-level care provision, education, income, and wealth, and means of country-level indicators by country and total sample. Sources: ^a^SHARE (waves 1, 2, 4, 5, 6) and ELSA (waves 2, 3, 4, 6, 7), ^b^UNU-WIDER, ^c^Eurostat

	**North**		**West**									**South**				**East**						
	DK	SE	AT	BE	GB	IE	FR	DE	NL	LU	CH	PT	ES	IT	GR	HR	CZ	EE	HU	PL	SI	Total
% care provision	5.1	3.9	6.7	8.1	6.1	8.8	7.0	6.0	5.4	7.0	4.9	11.6	9.0	8.8	8.2	8.5	8.8	9.4	9.6	9.9	7.2	7.4
*Individual-level*^*a*^* socioeconomic indicators*																						
% *Education*																						
Medium	37.6	32.7	52.5	29.8	54.0	18.4	40.5	54.4	27.6	42.3	63.6	12.0	13.0	26.8	27.9	31.0	50.3	52.9	57.7	53.1	55.0	40.6
Low	8.9	30.4	12.8	30.2	21.8	28.1	32.8	5.3	37.6	32.6	13.3	75.2	73.4	61.9	46.7	46.4	31.6	16.3	19.7	34.6	23.7	32.0
High	53.5	37.0	34.7	40.0	24.3	53.5	26.7	40.3	34.8	25.1	23.0	12.8	13.6	11.3	25.4	22.6	18.1	30.8	22.6	12.4	21.3	27.5
% *Couple income*																						
Middle medium income	47.9	48.0	40.0	31.8	40.9	25.7	36.7	37.1	35.1	32.9	35.7	24.4	33.5	34.6	32.9	29.6	46.5	44.9	40.8	37.2	33.9	37.9
Poor	6.7	6.6	13.6	14.6	10.9	22.7	14.3	14.3	14.3	16.2	15.2	31.2	16.9	19.9	20.1	24.8	14.5	15.9	17.9	20.3	19.9	15.3
Low medium income	32.9	36.1	31.3	31.6	33.1	21.9	33.4	32.5	30.5	31.7	29.2	17.6	28.6	28.0	28.8	24.5	28.9	25.2	31.0	29.6	26.1	30.1
High medium income	10.0	7.5	9.4	9.5	10.2	10.4	9.5	10.2	8.4	10.2	10.3	8.6	11.6	9.9	11.1	12.2	7.5	9.3	7.5	8.4	8.4	9.6
High income	2.6	1.7	5.7	12.5	4.9	19.4	6.1	6.1	11.6	9.0	9.5	18.3	9.4	7.6	7.1	8.9	2.8	4.7	2.8	4.5	11.7	7.1
% *Household wealth (Euros)*																						
0 to 49 999	9.2	12.9	27.5	10.6	9.2	6.8	12.6	24.5	23.5	7.3	15.1	27.9	13.6	16.3	20.0	39.7	40.9	46.3	65.0	68.6	22.8	22.2
Debt	3.9	3.8	2.3	1.6	3.9	1.7	1.9	3.7	3.2	2.2	3.2	3.4	1.8	1.7	3.7	3.1	2.6	1.1	2.5	3.7	1.4	2.6
50 000 to 99 999	7.8	12.7	10.7	5.1	3.7	2.0	6.3	11.1	6.7	1.5	6.8	19.3	14.6	9.4	24.5	25.1	28.8	22.2	21.8	19.6	17.7	12.7
100 000 and more	79.1	70.6	59.4	82.7	83.2	89.6	79.3	60.7	66.7	89.0	74.9	49.5	70.0	72.5	51.9	32.1	27.8	30.4	10.7	8.2	58.1	62.6
*Country level*																						
Mean Gini coefficient^b^	26.8	27.4	30.5	28.5	34.8	32.0	31.8	30.9	29.0	32.4	32.3	36.3	35.1	34.5	34.8	31.1	26.4	32.5	29.2	33.8	25.3	31.2
Mean Social Expenditure, %GDP^c^	30.9	29.0	27.9	27.4	25.5	16.8	29.9	27.8	25.9	22.5	23.5	24.3	23.1	26.4	22.0	21.4	18.7	15.7	21.3	18.2	23.9	24.7
Sample size	4234	5146	4197	7132	10,374	768	5681	6792	5071	1663	3352	1823	6993	6719	4700	2054	6253	5761	2412	2430	4317	97,872

International Organization for Standardization (ISO) 3166 alpha-2 country codes: North includes DK—Denmark, SE—Sweden. West includes AT—Austria, BE—Belgium, GB—Great Britain used as substitute for England, and IE—Ireland, DE—Germany, FR—France, NL—the Netherlands, LU—Luxembourg, and CH—Switzerland. South includes PT—Portugal, ES—Spain, IT—Italy, and GR—Greece. East includes HR—Croatia, CZ—Czech Republic, EE—Estonia, HU—Hungary, PL—Poland, and SI—Slovenia

Care provision is a binary variable. Normally, the use of logistic regression models is advisable with dichotomous outcome variables. However, research has shown that logistic regression models are not ideal for comparison across models and groups (Allison 1999; Mood 2010). While several methodological approaches can address this limitation (see for example Breen et al. 2012), we find the usage of Poisson models as proposed by Barros and Hirakata (2003) to be most advantageous. Poisson models are widely used for the analysis of count or zero-inflated variables. However, it is also possible to use them in the context of binary data analysis with the advantage that the well-known problems of logistic regression analysis do not apply to Poisson models.

We examined the association between both individual and country-level socioeconomic inequalities and care provision within the household. Therefore, we used two-level random intercept multilevel Poisson regression model with individuals nested within countries (see Rabe-Hesketh and Skrondal 2008 for details on different types of multilevel models). We first estimated an empty model (M1) that contained only the constant and the macro-level error terms to determine the variation in care provision on household and country level. Then we examined the main associations between individual socioeconomic status (education, income, and wealth) and informal care provision within the household (M2), followed by a model that also included individual-level control variables (M3). Next, we included country-level indicators, one at a time: Gini coefficient (M4) and public social expenditure (M5).

## Results

### Descriptive results

Table 2 provides a description of the sample and the differences between within-household caregivers and non-caregivers in the individual-level variables included in our multivariate models. Relative to non-caregivers, caregivers were more likely to have lower levels of education, income (poor to low middle income), and wealth. Caregivers were also more likely to be older, female, living in larger households, not working, and to report poorer health relative to their non-caregiving counterparts.

Table 3 presents the distribution of care provision within the household, individual socioeconomic resources (education, income, and wealth), and country-level indicators across the 21 countries in our study. The average prevalence of care provision inside the household was 7% but varied cross-nationally from the lowest observed in Sweden (4%) to the highest in Portugal (12%). Several Southern and Eastern European countries also showed high prevalence of care provision within the household including Croatia, Italy, Czech Republic, Spain, Hungary, and Poland (9 to 10%).

Regarding macro-level socioeconomic resources, income inequality varied within regions as some Northern and Western countries showed relatively high levels (e.g., Luxembourg, France, Switzerland, and England with an average Gini index of 32 to 35) while other Northern and Western countries had relatively low income inequality (e.g., Sweden, Denmark, the Netherlands with an average Gini index of 27 to 29). Portugal showed the highest income inequality with an average Gini index of 36. Finally, average social expenditure as a percentage of GDP ranged from a low of 16 to 18 percent in Estonia, Ireland, Czech Republic, and Poland to 31 percent in Denmark.

### Multivariate results

Table 4 presents the results of our multivariate analyses of care provision within the household. M2 and M3 examined the association between individual-level socioeconomic resources and caregiving within the household, without (M2), and with (M3) individual-level covariates. Our unadjusted model, M2, showed that low education and income (specifically low medium income) were positively associated, whereas higher levels of education and income were negatively associated, with the incidence of caregiving within the household. Regarding wealth differences, wealthier households (50,000 or more Euros) showed a lower incidence of caregiving within the household relative to less wealthy households. In brief, providing informal care was more common among individuals with lower relative to higher SES, and this finding held for all SES indicators.

**Table 4:** Multilevel Poisson regression incidence rate ratios of care provision within the household (n (individuals) = 97,872, n (countries) = 21). Source: SHARE (1, 2, 4, 5, 6) and ELSA (2, 3, 4, 6, 7); *** p < 0.001, ** p < 0.01, * p < 0.05; standard errors in parentheses

Variables	M1	M2	M3	M4	M5
*Individual level*					
*Household education (medium)*					
Low		1.22***	1.06	1.05	1.06
		(0.04)	(0.03)	(0.03)	(0.03)
High		0.92*	0.93*	0.93*	0.93*
		(0.03)	(0.03)	(0.03)	(0.03)
*Couple income (middle medium income)*					
Poor		1.05	0.95	0.95	0.96
		(0.04)	(0.04)	(0.04)	(0.04)
Low medium income		1.21***	1.13***	1.13***	1.13***
		(0.03)	(0.03)	(0.03)	(0.03)
High medium income		0.75***	0.82***	0.82***	0.82***
		(0.04)	(0.04)	(0.04)	(0.04)
High income		0.93	1.01	1.01	1.01
		(0.05)	(0.05)	(0.05)	(0.05)
*Household wealth (0—49,999 Euros)*					
Debts		0.98	1.08	1.08	1.08
		(0.07)	(0.08)	(0.08)	(0.08)
50′000–99′999		0.84***	0.89**	0.89**	0.89**
		(0.03)	(0.03)	(0.03)	(0.03)
100′000 +		0.74***	0.78***	0.78***	0.79***
		(0.02)	(0.02)	(0.02)	(0.02)
Age			1.02***	1.02***	1.02***
			(0.00)	(0.00)	(0.00)
*Gender (Men)*					
Women			1.33***	1.33***	1.33***
			(0.03)	(0.03)	(0.03
Household size			1.13***	1.13***	1.13***
			(0.01)	(0.01)	(0.01)
*Employment status (not working)*					
Full-time			0.76***	0.76***	0.76***
			(0.03)	(0.03)	(0.03)
Part-time			0.76***	0.76***	0.77***
			(0.04)	(0.04)	(0.04)
*IADL limitations (none)*					
At least 1 limitation			1.43***	1.43***	1.43***
			(0.04)	(0.04)	(0.04)
*Interview year (2004–2005)*					
2006–2007			1.13**	1.13**	1.12*
			(0.05)	(0.05)	(0.05)
2008–2012			1.24***	1.24***	1.23***
			(0.05)	(0.05)	(0.05)
2013–2015			1.25***	1.26***	1.25***
			(0.05)	(0.05)	(0.05)
*Country level*					
Gini coefficient				1.03**	
				(0.01)	
Social expenditure, %GDP					0.98*
					(0.009)
Constant	0.07***	0.08***	0.02***	0.01***	0.03***
	(0.00)	(0.01)	(0.00)	(0.00)	(0.01)
*Variance and model fit*					
Country level	0.07**	0.04**	0.03**	0.02**	0.02**
	(0.02)	(0.02)	(0.01)	(0.01)	(0.01)
AIC	51,674.1	51,270.9	50,505.7	50,501.8	50,503.8
BIC	51,693.1	51,375.3	50,695.6	50,701.1	50,703.1
Log likelihood	−25,835.07	−25,624.47	−25,232.87	−25,229.9	−25,230.89
Likelihood ratio test		421.19***	783.21***	5.93**	3.95*

Likelihood ratio tests M1 nested in M2; M2 nested in M3; M3 nested in M4: M3 nested in M5

Adjusting for individual-level covariates (M3) did not change the directions but attenuated the strength of the associations between individual-level socioeconomic resources (education, income, and wealth) and care provision within the household. The addition of covariates significantly improved the model and was a better fit for the data based on the statistically significant likelihood ratio tests as well as the AIC and BIC statistics. Regarding our sociodemographic covariates, we found informal care provision to be positively associated with age, household size, and women were more likely than men to provide informal care within the household. Employment, full-time and part-time (relative to not working), was negatively associated with informal care provision. Regarding respondents’ health, we found that having one or more limitations with instrumental activities of daily living was positively associated with informal care provision. While having limitations with instrumental activities of daily living could imply that these older adults may also need help themselves, our finding suggested that these respondents might be reciprocating care they received either currently or in the past or were surrounded by people with care needs as network members typically have similar characteristics.

M4 and M5 show the associations between country-level characteristics and care provision within the household. Accounting for the country’s level of income inequality (M4) as measured by the Gini coefficient did significantly improve model fit, compared to M3. It was positively associated with care provision within the household. Furthermore, the direction and strength of associations between individual-level income and wealth, and care provision within the household as observed in M3, were maintained. Additional analyses, not shown, indicated that even after controlling for the country’s overall wealth, the direction and strength of the association between within-country income inequality and care provision within the household were unchanged. Thus, countries with higher levels of income inequality, on average, showed a slightly higher incidence of care provision within the household. The inclusion of the country’s level of social expenditure (M5) also significantly improved model fit compared to M3. Results showed that countries with higher levels of social expenditure, on average, exhibited a slightly lower incidence of care provision within the household. Thus, our findings suggested that socioeconomic inequalities at the macro-level were associated with an increased propensity for older adults’ provision of informal care, whereas higher social protection expenditure within countries was associated with a lower propensity for older adults’ informal care provision.

Across all models, the estimated country-level variance indicated there was significant between-country variation in care provision within the household among the countries included in our study. Notably, the addition of our macro-indicators (Gini coefficient and social spending as a % of GDP) substantially decreased the AIC and BIC (e.g., 1173 and 992 in M4, respectively when accounting for income inequality), compared to the empty model. Thus, the country-level indicators improve predictive power and explain some of the cross-national variation in care provision within the household. Yet, the variance remained statistically significant suggesting additional unobserved heterogeneity in informal care provision across countries.

## Discussion

As European societies age, increasing care demands put formal and informal care systems under stress. While some research has suggested that older adult caregivers have better health outcomes than non-caregivers (Bertrand et al. 2012), and caregiving can enhance caregivers’ self-esteem and relationships (Doris et al. 2018; Raschick and Ingersoll-Dayton, 2004), other research has shown that care provision within the household is especially detrimental to informal caregivers’ health (Kaschowtiz and Brandt 2017). This study set out to reveal whether informal care provision to vulnerable household members falls disproportionally on lower socioeconomic individuals. Such knowledge is critical to identifying individuals’ risks of potential caregiving burden and needs of targeted support. Moreover, studying how socioeconomic resources at the national-level structure individuals’ informal care provision to household members is also important for identifying which types of investments may be helpful to reduce strains, which often come with providing informal care to someone in the household. Motivated by these concerns, this study examined how individual socioeconomic resources, national levels of social inequality and social spending were related to informal care provision within the household.

Overall, our results showed that lower individual socioeconomic resources—education, income, and wealth—were associated with a higher incidence of informal care provision within the household. Importantly, our study showed socioeconomic inequalities irrespective of the SES measurement suggesting that educational, income and wealth inequalities in care provision exist simultaneously. This study therefore provided strong evidence for the existence of socioeconomic inequality in informal care provision inside the household with lower strata being more likely, and higher strata less likely, to provide care. Our findings align with previous studies on informal care *use*, which generally conclude that socioeconomically disadvantaged households are more heavily reliant on informal care (Floridi et al. 2020; Rodrigues et al. 2018). As informal care can be costly and caregivers, especially women, often reduce work hours or exit the labor market (Moussa 2019), the higher incidence of informal caregiving among socioeconomically disadvantaged individuals potentially increases economic insecurity while widening socioeconomic and gender inequalities.

We also examined whether socioeconomic inequality at the national level is related to individuals’ informal care provision. We observed that higher national income inequality was associated with a higher incidence of informal care provision within the household. A possible interpretation of this finding is that income inequality can strengthen family solidarity; at least where family members have demanding personal care needs. Income inequality arguably reduces social cohesion and social contact, which can inadvertently increase individuals’ reliance on close-knit family ties during vulnerable periods of their lives. Another interpretation is that societies with higher income inequality may have larger proportions of their population to be in need of care, driving up levels of informal care, since higher levels of income inequality are also associated with poor population health and other social strains (Wilkinson and Pickett 2009).

Our findings underscore the importance of social spending for older adults’ provision of informal care. We found that higher social spending was negatively associated with older adults providing informal care within their households, which likely refers to intensive care. This negative association is consistent with previous European studies (Brandt et al. 2009; Verbakel 2018) that suggest the specialization of state and family-based support as it relates to intensive caregiving. Arguably, higher state support for vulnerable members of society can relieve individuals of their responsibility to provide demanding care tasks (as is typical in personal caregiving) but continue to provide less intensive and voluntary support. Our findings suggest that older adults in countries with limited social spending possibly face higher strains in supporting their vulnerable family members, although our study admittedly did not examine the relationship between social spending and alternative forms of support (e.g., household help) *and* social spending does not guarantee high quality or supportive services. The negative association between social spending and within-household informal care provision may also reflect the care recipients’ (and caregivers’) preferences for non-familial support under circumstances that require regular personal caregiving. Social policy contexts partially structure cultural expectations about family care, individuals’ care preferences, and caregiving decisions (Mair et al. 2016). Furthermore, wealthier countries at more advanced stages of population aging potentially have stronger welfare infrastructure to meet the increasing health and care needs of their aging population relative to less developed and advanced aging countries (He et al. 2016). Therefore, differences in the stage of demographic transition across the countries can influence social policy and individuals’ care arrangements. We welcome future multilevel studies to examine and disentangle the role of several macro-level factors—demographic, cultural, economic, policy—for individual informal care provision within the household.

While our study offers unique and timely contributions, we acknowledge there are a few limitations. First, our analysis utilizes cross-sectional data. Therefore, we cannot address issues of reverse causality between informal care provision and individual socioeconomic resources (e.g., informal care provision can lead to lower income). Second, we are unable to examine the intensity (e.g., number of hours) of caregiving, which is also likely to vary by individuals’ locations in the socioeconomic strata and their national contexts. Third, our study focuses on care provision within the household, broadly, and does not differentiate health statuses of the care recipients (e.g., dementia), and whether care provision can be shared with other informal or formal caregivers. Although within-household informal caregiving is generally more time-intensive and burdensome (Pristavec 2019), still variation between situations will exist that we cannot detect. Individual and contextual socioeconomic resources may be more or less salient depending on the recipients’ care needs.

Despite these limitations, our study advances and contributes to the existing research on informal caregiving by examining the degree of socioeconomic inequality in older Europeans’ informal care provision within their households and the impact of societal income inequality and social spending on informal care provision. Informal caregiving, especially within the household, is often burdensome and costly, which can contribute to health and economic vulnerabilities as people age. As socioeconomically disadvantaged individuals are more likely to provide informal care to a household member, they are also more vulnerable to caregiving-related health and financial vulnerabilities. Moreover, our country-level socioeconomic indicators provide modest preliminary evidence that income inequality and welfare state structures are associated with the likelihood of providing care to household members, possibly through shaping older adults’ perceptions of choice, and likely preferences, to be active care providers for their ailing family members. Thus, having a more equitable distribution of socioeconomic resources and increasing social spending to better support individuals and households, which may be unable to support themselves, may minimize intensive caregiving and benefit older adults as they age.
